# Comparative genomic analysis of the genus *Staphylococcus *including *Staphylococcus aureus *and its newly described sister species *Staphylococcus simiae*

**DOI:** 10.1186/1471-2164-13-38

**Published:** 2012-01-24

**Authors:** Haruo Suzuki, Tristan Lefébure, Paulina Pavinski Bitar, Michael J Stanhope

**Affiliations:** 1 Department of Population Medicine and Diagnostic Sciences, College of Veterinary Medicine, Cornell University, Ithaca, NY 14853, USA; 2 Université de Lyon; UMR5023 Ecologie des Hydrosystèmes Naturels et Anthropisés; Université Lyon 1; ENTPE; CNRS; 6 rue Raphaël Dubois, 69622 Villeurbanne, France

## Abstract

**Background:**

*Staphylococcus *belongs to the Gram-positive low G + C content group of the Firmicutes division of bacteria. *Staphylococcus aureus *is an important human and veterinary pathogen that causes a broad spectrum of diseases, and has developed important multidrug resistant forms such as methicillin-resistant *S. aureus *(MRSA). *Staphylococcus simiae *was isolated from South American squirrel monkeys in 2000, and is a coagulase-negative bacterium, closely related, and possibly the sister group, to *S. aureus*. Comparative genomic analyses of closely related bacteria with different phenotypes can provide information relevant to understanding adaptation to host environment and mechanisms of pathogenicity.

**Results:**

We determined a Roche/454 draft genome sequence for *S. simiae *and included it in comparative genomic analyses with 11 other *Staphylococcus *species including *S. aureus*. A genome based phylogeny of the genus confirms that *S. simiae *is the sister group to *S. aureus *and indicates that the most basal *Staphylococcus *lineage is *Staphylococcus pseudintermedius*, followed by *Staphylococcus carnosus*. Given the primary niche of these two latter taxa, compared to the other species in the genus, this phylogeny suggests that human adaptation evolved after the split of *S. carnosus*. The two coagulase-positive species (*S. aureus *and *S. pseudintermedius*) are not phylogenetically closest but share many virulence factors exclusively, suggesting that these genes were acquired by horizontal transfer. Enrichment in genes related to mobile elements such as prophage in *S. aureus *relative to *S. simiae *suggests that pathogenesis in the *S. aureus *group has developed by gene gain through horizontal transfer, after the split of *S. aureus *and *S. simiae *from their common ancestor.

**Conclusions:**

Comparative genomic analyses across 12 *Staphylococcus *species provide hypotheses about lineages in which human adaptation has taken place and contributions of horizontal transfer in pathogenesis.

## Background

*Staphylococcus *belongs to the Gram-positive low G + C content group of the Firmicutes division of bacteria. *Staphylococcus aureus *is an important human and veterinary pathogen that causes a broad spectrum of diseases, and has developed important multidrug resistant forms such as methicillin-resistant *S. aureus *(MRSA) and vancomycin-resistant *S. aureus *(VRSA) [1-3]. Despite emergence of MRSA in human and various animal species, mechanisms of host adaptation are poorly understood [4]. Comparative genomic analyses of phylogenetically closely related bacteria with different phenotypes (e.g. host specificity and pathogenicity) can provide information relevant to understanding adaptation to host environment and mechanisms of pathogenicity [5-10]. *Staphylococcus simiae *was isolated from South American squirrel monkeys in 2000, and is a coagulase-negative bacterium closely related, and indeed possibly the sister group, to *S. aureus *[11]. Comparison between *S. aureus *and *S. simiae *genomes could provide valuable information regarding host adaptation and pathogenesis. Thus, we determined a draft genome sequence of *S. simiae *type strain CCM 7213T (= LMG 22723T), and included it in comparative genomic analyses with 11 other *Staphylococcus *species.

## Methods

### Genome sequencing and data collation

We determined the genome sequence of *Staphylococcus simiae *type strain CCM 7213T (= LMG 22723T), isolated from the faeces of a South American squirrel monkey [11]. Roche/454 pyrosequencing, involving a single full run of the GS-20 sequencer, was used to determine the sequence of the *Staphylococcus simiae *genome. The sequences were assembled (*De novo *assembly with Newbler Software) into 565 contigs. Genome annotation for the strain was done by the NCBI Prokaryotic Genomes Automatic Annotation Pipeline. The *S. simiae *whole genome shotgun project has been deposited at DDBJ/EMBL/GenBank under the accession AEUN00000000. The version described in this paper is the first version, AEUN01000000. For comparative analysis genome sequences of bacteria in GenBank format [12] were retrieved from the National Center for Biotechnology Information (NCBI) site ftp://ftp.ncbi.nlm.nih.gov/. We analyzed sequences of 28 *Staphylococcus *strains belonging to 12 different species, and an outgroup *Macrococcus caseolyticus *JCSCS5402 [13] (Table 1 and Additional file 1, Table S1). The 16 *Staphylococcus aureus *strains included COL [14], ED133 [15], ED98 [16], JH1, JH9, MRSA252 [17], MSSA476 [17], Mu3 [18], Mu50 [19], MW2 [20], N315 [19], NCTC_8325, Newman [21], RF122/ET3-1 [22], USA300_FPR3757 [23], and USA300_TCH1516 [24]. The remaining 12 *Staphylococcus *strains included *Staphylococcus capitis *SK14 [25], *Staphylococcus caprae *C87, *Staphylococcus carnosus *TM300 [26], *Staphylococcus epidermidis *ATCC 12228 [27], *Staphylococcus epidermidis *RP62a [14], *Staphylococcus haemolyticus *JCSC1435 [28], *Staphylococcus hominis *SK119, *Staphylococcus lugdunensis *HKU09-01 [29], *Staphylococcus pseudintermedius *HKU10-03 [30], *Staphylococcus saprophyticus *ATCC_15305 [31], *Staphylococcus simiae *[11], and *Staphylococcus warneri *L37603. Genome sequence analyses were implemented using Bioperl version 1.6.1 [32] and G-language Genome Analysis Environment version 1.8.12 [33-35]. Statistical tests and graphics were implemented using R, version 2.11.1 [36].

**Table 1:** Genomic features of *Macrococcus caseolyticus* and 28 *Staphylococcus* strains.

Organism	Size (bp)	%G + C	*S*	No.CDS	No.MCL
*Macrococcus caseolyticus *JCSC5402	2219737	36.6	1.27	2052	1688
*Staphylococcus aureus *COL	2813862	32.8	1.58	2615	2304
*Staphylococcus aureus *ED133	2832478	32.9	1.55	2653	2291
*Staphylococcus aureus *ED98	2847542	32.8	1.56	2689	2338
*Staphylococcus aureus *JH1	2936936	32.9	1.40	2780	2389
*Staphylococcus aureus *JH9	2937129	32.9	1.40	2726	2389
*Staphylococcus aureus *MRSA252	2902619	32.8	1.57	2650	2353
*Staphylococcus aureus *MSSA476	2820454	32.8	1.57	2590	2330
*Staphylococcus aureus *Mu3	2880168	32.9	1.54	2690	2368
*Staphylococcus aureus *Mu50	2903636	32.8	1.54	2730	2389
*Staphylococcus aureus *MW2	2820462	32.8	1.58	2624	2319
*Staphylococcus aureus *N315	2839469	32.8	1.55	2614	2307
*Staphylococcus aureus *NCTC_8325	2821361	32.9	1.56	2891	2347
*Staphylococcus aureus *Newman	2878897	32.9	1.54	2614	2338
*Staphylococcus aureus *RF122	2742531	32.8	1.55	2509	2267
*Staphylococcus aureus *USA300_FPR3757	2917469	32.7	1.58	2604	2385
*Staphylococcus aureus *USA300_TCH1516	2903081	32.7	1.56	2689	2382
*Staphylococcus capitis *SK14	2435835	32.8	1.47	2230	1847
*Staphylococcus caprae *C87	2473608	32.6	1.46	2402	1887
*Staphylococcus carnosus *TM300	2566424	34.6	1.42	2461	1859
*Staphylococcus epidermidis *ATCC_12228	2564615	32.0	1.12	2482	1972
*Staphylococcus epidermidis *RP62A	2643840	32.1	1.15	2525	2068
*Staphylococcus haemolyticus *JCSC1435	2697861	32.8	1.42	2692	2021
*Staphylococcus hominis *SK119	2226236	31.3	1.53	2182	1729
*Staphylococcus lugdunensis *HKU09-01	2658366	33.9	1.26	2490	1896
*Staphylococcus pseudintermedius *HKU10-03	2617381	37.5	1.50	2450	1910
*Staphylococcus saprophyticus *ATCC_15305	2577899	33.2	1.34	2514	1838
*Staphylococcus simiae *CCM_7213	2587121	31.9	1.33	2592	1950
*Staphylococcus warneri *L37603	2425653	32.8	1.42	2381	1875

%G + C = 100 × (G + C)/(A + T + G + C).

*S *= Selected codon usage bias.

No.CDS = Number of protein-coding sequences.

No.MCL = Number of protein families built by BLAST and Markov clustering.

### Gene content analysis

Protein-coding sequences were retrieved from chromosomes and plasmids of the 29 strains of bacteria (Table 1 and Additional file 1 Table S1). A group of homologous proteins (protein family) was built by all-against-all protein sequence comparison of the 29 strains' proteomes using BLASTP [37], followed by Markov clustering (MCL) with an inflation factor of 1.2 [38]. Homologous proteins were identified by BLASTP on the criteria of an E-value cutoff of 1e-5, and minimum aligned sequence length coverage of 50% of a query sequence. This approach yielded 5014 protein families containing 74122 individual proteins from the 29 strains (see Additional file 1, Table S2). We assigned functions to each protein family by using multiple databases: the Clusters of Orthologous Groups (COG) [39,40], JCVI [41], KEGG [42], SEED [43], Virulence Factors Database (VFDB) [44], MvirDB [45], Pfam [46], and Gene Ontology (GO) [47] database. We searched protein sequences against the Pfam library of hidden Markov models (HMMs) using HMMER http://hmmer.janelia.org/, and converted Pfam accession numbers to GO terms using the 'pfam2go' mapping http://www.geneontology.org/external2go/pfam2go. We performed TBLASTN searches (on the criteria of an E-value cutoff of 1e-5, and minimum aligned sequence length coverage of 50% of a query sequence) of each of the 29 strains' proteomes against whole nucleotide sequences of all the other strains to avoid artefacts caused by differences in protein-coding sequence prediction [8,48]. The resulting gene content (binary data for presence or absence of each protein family) is shown in Additional file 1, Table S2.

Hierarchical clustering (UPGMA) of the 29 strains was performed using a distance between two genomes based on gene content (binary data for presence or absence of each protein family) measured by one minus the Jaccard coefficient (Jaccard distance). To identify taxon-specific genes, we calculated Cramer's V to screen protein families showing biased distributions between comparative groups. Cramer's V is a measure of the degree of correlation in contingency tables. Cramer's V values close to 0 indicate weak associations between variables, while those close to 1 indicate strong associations. We used the most stringent threshold (i.e. Cramer's V of 1) to identify *S. aureus *and *S. simiae *unique proteins or protein families. To examine over- or underrepresented functional categories in the 16 *S. aureus *strains relative to the single *S. simiae *strain, a 2 × 2 contingency table was constructed for each functional category from the COG, JCVI, KEGG, SEED, VFDB, and GO databases: (a) the number of *S. aureus *protein families in this category; (b) the number of *S. aureus *protein families not in this category; (c) the number of *S. simiae *protein families in this category; and (d) the number of *S. simiae *protein families not in this category. The odds ratio (= ad/bc) was used to rank the relative over-representation (> 1) or under-representation (< 1) of each of the functional categories.

### Phylogenetic analysis

Of the 5014 protein families, 497 were shared by all the 29 strains and contained only a single copy from each strain (did not contain paralogs). This set of 497 single-copy core genes were identified as putative orthologous genes. The sequences were first aligned at the amino acid level using Probalign [49], then backtranslated to DNA. Alignment columns with a posterior probability < 0.6 were removed, and alignments with > 50% of the sites removed were discarded from the analysis. Multiple alignments with Probalign retained 491 reliably aligned genes from a set of the 497 orthologous genes. Gene trees were reconstructed using PhyML (Phylogenetic estimation using Maximum Likelihood) [50,51] with the General Time Reversible plus Gamma (GTR + G) substitution model of DNA evolution, and the Subtree Pruning-Regrafting (SPR) branch-swapping method. Each gene tree search was bootstrapped (500 pseudoreplicates) using PhyML with the Nearest-Neighbor Interchange (NNI) branch-swapping method to detect genes that support or conflict with various bipartitions. A majority rule consensus of the gene trees was constructed using the consense program of PHYLIP 3.69 [52]. All the alignments were also concatenated, and a tree search was performed using PhyML with the same settings as for the gene trees. *Macrococcus caseolyticus *JCSCS5402 was used as an outgroup to root the trees. We used DendroPy [53] to annotate the nodes of the estimated consensus and concatenated gene trees with the percentage of gene trees in which the node was found. Resulting phylogenetic trees were drawn using the R package APE (Analysis of Phylogenetics and Evolution) [54].

## Results and discussion

### Genomic features

Roche/454 pyrosequencing was used to determine the sequence of the *Staphylococcus simiae *genome. A total of 643168 single-end reads resulted from the GS-20 sequencer for *S. simiae*. *De novo *assembly with Newbler yielded 565 contigs for a total genome size of 2,587,121 bp with G + C content of 31.9% and 2592 protein-coding sequences (Table 1) with sequencing coverage of 27.4 (2623 singleton reads). The N50 size of the contigs is 19200.

Genome size was larger in *S. aureus *(ranging from to 2.743 Mbp to 2.937 Mbp) than in the other *Staphylococcus *species (ranging from to 2.220 Mbp to 2.698 Mbp). Genomic G + C content of *M. caseolyticus *(36.6%), *S. pseudintermedius *(37.5%), and *S. carnosus *(34.6%) were higher than those of the other *Staphylococcus *species (ranging from to 31.3% to 33.9%). Genomic G + C content is a result of mutation and selection [55], involving multiple factors including environment [56], symbiotic lifestyle [57], aerobiosis [58], and nitrogen fixation ability [59]. Bacteria showing evidence of translational selection on synonymous codon usage of highly expressed genes tend to have more rRNA operons, more tRNA genes, and faster growth rate [60]. The strength of translationally selected codon usage bias (*S*) [60] was significantly higher in *S. aureus *(median *S *= 1.56) than in the other *Staphylococcus *species (median *S *= 1.42) based on Mann-Whitney test (*P *< 10^-4^); *S. epidermidis *strains RP62A (*S *= 1.15) and ATCC_12228 (*S *= 1.12) showed the lowest values of *S *(Table 1).

### Phylogeny

**Figure 1:**
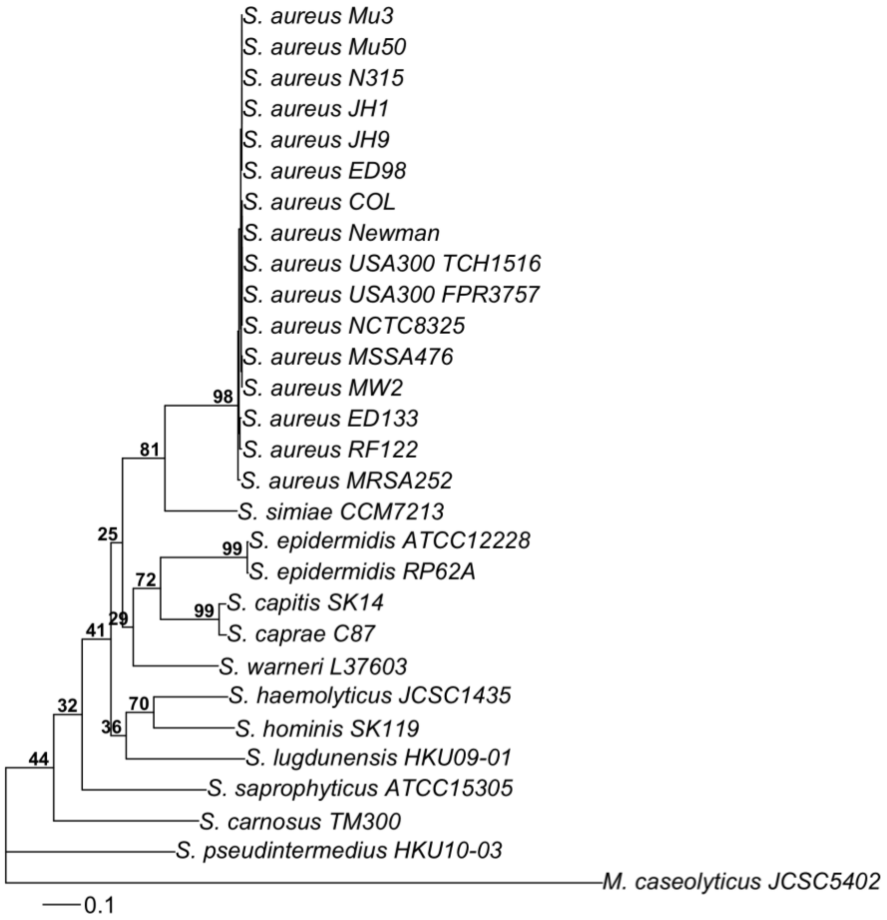
**Phylogenetic tree inferred from concatenated genes**. Maximum likelihood tree obtained from a concatenated nucleotide sequence alignment of the orthologous core genes for the 28 *Staphylococcus *strains and *Macrococcus caseolyticus *JCSCS5402 (outgroup). The horizontal bar at the base of the figure represents 0.1 substitutions per nucleotide site. The percentages of genes that support the branches of the tree are indicated.

The 491 orthologous genes were used to infer phylogenetic relationships of the 28 *Staphylococcus *strains. The phylogenetic tree inferred from concatenated genes (Figure 1), as well as the majority rule consensus of the individual gene trees (Additional file 2, Figure S1) demonstrated that the vast majority of genes supported the monophyly of the 16 *S. aureus *strains (98%), the monophyly of the two *S. epidermidis *strains (99%), and the monophyly of the clade of *S. aureus *and *S. simiae *(81%), supporting previous suggestions that *S. simiae *is the putative sister group to *S. aureus *[11]. Of the 491 gene trees, 486, 491, and 322 (99%, 100%, and 65.6%) supported these three nodes with bootstrap support in excess of 70%, and none had a strongly supported conflicting signal compared to that topology.

The most basal *Staphylococcus *lineage in our phylogenetic trees was *S. pseudintermedius*, followed by *S. carnosus*. Although support for these two nodes involved only 217 and 156 (44% and 32%) of the 491 gene trees, there were only a few instances of genes that had a strongly supported conflicting signal compared to that topology. Only 37 and 17 (7.5% and 3.5%) of the 491 genes had a conflicting evolutionary history for these two nodes with bootstrap support in excess of 70%, while 107 and 56 (21.8% and 11.4%) supported these two nodes with bootstrap support in excess of 70%. *Staphylococcus *species which are indigenous to humans include *S. aureus*, *S. epidermidis*, *S. caprae*, *S. capitis*, *S. warneri*, *S. hominis*, *S. haemolyticus*, *S. lugdunensis*, and *S. saprophyticus *[61]. *S. carnosus *has not been isolated from human skin or mucosa, and its natural habitat is unknown despite its natural occurrence in meat and fish products [26]. *S. pseudintermedius *is a coagulase-positive species from animals [62], and *M. caseolyticus *is typically isolated from animal skin and food such as milk and meat [13]. Although species indigenous to animals may be found occasionally on humans by recent contact [61,63], our phylogeny suggests that human adaptation evolved after the split of *S. carnosus*.

### Gene content

**Figure 2:**
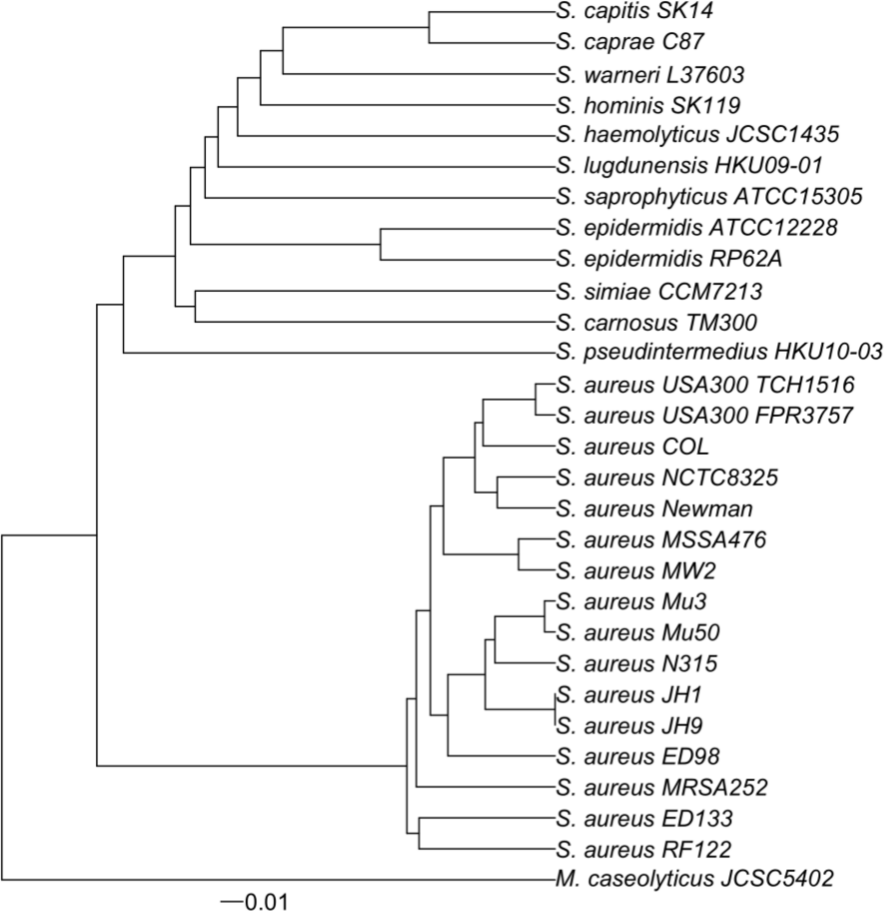
**Gene content dendrogram**. A dendrogram constructed by hierarchical clustering based on dissimilarities in gene content (binary data for presence or absence of protein families) for the 28 *Staphylococcus *strains and *Macrococcus caseolyticus *JCSCS5402. The dissimilarities were measured using the Jaccard distance, ranging from 0 to 1, represented by the horizontal bar at the base of the figure.

The 69171 protein-coding sequences from the 29 strains were classified into 5361 homolgous groups or protein families (see Additional file 1, Table S2). A dendrogram constructed by hierarchical clustering (Figure 2) indicates that the overall similarity of the 29 strains based on gene content (binary data for presence or absence of different protein families) did not strictly follow their phylogenetic history (Figure 1 and Additional file 2, Figure S1). This indicates that the *Staphylococcus *gene repertoire reflects not only vertical inheritance of genes, but probable instances of one or more of the following: lineage-specific gene loss, non-orthologous gene displacement, or gene gain through horizontal gene transfer [64].

We assessed presence of virulence factors in the *Staphylococcus *strains based on the gene content table (Additional file 1, Table S2) and percent identity values of TBLASTN best hits against VFDB (Additional file 3, Figure S2). Many virulence genes of *S. aureus *are encoded on mobile genetic elements such as staphylococcal cassette chromosomes (SCC), genomic islands, pathogenicity islands, prophages, plasmids, insertion sequences, and transposons [2,3,65]. For movement, SCC carries cassette chromosome recombinase (*ccr*) gene(s) (*ccrAB *or *ccrC*) [66,67]. The three *ccr *genes (*ccrA*, *ccrB*, and *ccrC*) are homologous and have no homolog in *S. carnosus*. The genetic determinant of methicillin resistance (*mec*) is encoded on SCC in *S. aureus*, designated as SCC*mec *[68]. Expression of beta-lactamase (*blaZ*) and penicillin-binding protein 2a (PBP 2a) genes (*mecA*) is controlled by the BlaR-BlaI-BlaZ and MecR-MecI-MecA regulatory systems, respectively [69]. There is homology between *blaI *and *mecI*, between *blaR1 *and *mecR1*, and between the promoter and N-terminal portions of *blaZ *and *mecA *[70]. *mecA *gene homologs were present in all *Staphylococcus *species, while presence of *blaI*/*mecI *and *blaR1*/*mecR1 *gene homologs varied among different *Staphylococcus *species and even between different strains within *S. aureus*. *S. aureus *genomic islands and pathogenicity islands carry superantigenic toxic shock syndrome toxin-1 (TSST-1) encoded by *tst *[71] homologous to the staphylococcal exotoxin-like (*set*) proteins, renamed staphylococcal superantigen-like (*ssl*) proteins. The *tst *gene homolog was present in *S. carnosus *TM300 (Sca_0436 and Sca_0905) and *S. pseudintermedius *HKU10-03 (SPSINT_0099). A previous study [26] reported that *S. carnosus *TM300 lacks the known superantigens such as toxic shock syndrome toxin 1 (*tst*) and enterotoxins (*sea *to *sep*). The serine protease (*spl*) gene homolog was not found in *S. lugdunensis*. Lipoprotein (*lpl*) gene homologs were present in *S. aureus*, *S. epidermidis*, *S. haemolyticus*, and *S. lugdunensis*. *S. aureus *prophages carry virulence factors such as Panton-Valentine leukocidin (*lukS-PV *and *lukF-PV*), staphylokinase (*sak*), exfoliative toxin A (*eta*), and enterotoxins [72]. The *sak *gene homolog was present in the 12 *S. aureus *strains but absent in the 4 *S. aureus *strains (COL, ED133, ED98, and RF122). The *eta *gene homolog was present in *S. aureus*, *S. carnosus *TM300 (Sca_2302), and *S. pseudintermedius *HKU10-03 (SPSINT_0069). *S. aureus *can produce several homologous two-component pore-forming toxins including Panton-Valentine leukocidin (*lukS-PV *and *lukF-PV *on prophage), leukotoxin D and E (*lukD *and *lukE *on genomic island), and gamma-hemolysin (*hlgA*, *hlgB*, and *hlgC*) [73,74], with homologs present in *S. pseudintermedius *HKU10-03 (SPSINT_1566 and SPSINT_1567). Staphylococcal enterotoxins (*entD*, *entE*, *sea*, *seb*, *sec1*, *sec3*, *sed*, *seg2*, *seh*, and *sek2*) encoded on *S. aureus *mobile genetic elements [2] were homologous and have a single homolog in *S. pseudintermedius *HKU10-03 (SPSINT_0513). As expected, a secreted von Willebrand factor-binding protein (coagulase) [75] was present in the coagulase-positive staphylococci (*S. aureus *and *S. pseudintermedius*) but absent in the coagulase-negative staphylococci [76].

To identify *S. aureus *and *S. simiae *unique genes, we compared gene presence and absence between the 16 *S. aureus *strains and the other 12 *Staphylococcus *strains, and between the single *S. simiae *strain and the other 27 *Staphylococcus *strains. A total of 272 protein families were present in *S. aureus *but absent in the other *Staphylococcus *species (Additional file 1, Table S3). This set included known as well as candidate virulence factors of *S. aureus *such as staphylococcal complement inhibitor SCIN (fibrinogen-binding protein), hyaluronate lyase (*hysA*), GntR family transcriptional regulator, secretory extracellular matrix and plasma binding protein, *isdD *(Iron uptake; Heme uptake), zinc finger SWIM domain-containing protein, 1-phosphatidylinositol phosphodiesterase known as a virulence factor (Exoenzyme; Membrane-damaging; Phospholipase) of *Listeria monocytogenes *(serovar 1/2a) EGD-e, formyl peptide receptor-like 1 inhibitory protein, NADH dehydrogenase subunit, 3-methyladenine DNA glycosylase, probable exported proteins and membrane proteins. Genes encoding quaternary ammonium compound-resistance protein SugE were absent in *S. aureus *but present in the other *Staphylococcus *species. It was previously shown that high-level expression of SugE of *Escherichia coli *leads to resistance to a subset of toxic quaternary ammonium compounds [77]. A total of 129 unique protein families were present in *S. simiae *but absent in other S*taphylococcus *species (Additional file 1, Table S4). This set included surface anchored protein, DNA-3-methyladenine glycosylase II, reverse transcriptase, transcriptional regulators, and phage-related proteins. The *S. aureus *and *S. simiae *unique genes may have been gained on the branch leading to the *S. aureus *ancestor and the *S. simiae *strain, and could be linked to their specific host adaptation and pathogenesis. Many of these genes were, however, quite short (< 150 bp) and functionally unknown, and thus could be protein-coding sequence prediction error.

Enrichment tests across functional categories indicated that the JCVI mainrole categories "Cell envelope" (odds ratio = 1.15) and "Mobile and extrachromosomal element functions" (odds ratio = 1.38), the JCVI subrole categories "Pathogenesis" (odds ratio = 1.40) and "Prophage functions" (odds ratio = 1.38), the KEGG pathway map "*Staphylococcus aureus *infection" (odds ratio = 1.91), and the VFDB keyword "Type VII secretion system" (odds ratio = 7.06) were overrepresented in *S. aureus *relative to *S. simiae *(Additional file 1, Table S5). None of the functional categories were significantly over- or underrepresented based on Fisher's exact test after false discovery rate correction for multiple comparisons (*P *< 0.05). A total of 52 protein families associated with cell envelope were identified here, and the numbers were higher in *S. aureus *(ranging from 48 to 50) than in other *Staphylococcus *species (ranging from 33 to 45). Cell wall-associated proteins are involved in host-pathogen interactions, and those from *S. aureus *ED133 have been shown to be under diversifying selection pressure [15]. A total of 79 protein families associated with cell wall were identified here, and the numbers were higher in *S. aureus *(ranging from 60 to 64) than in other *Staphylococcus *species (ranging from 47 to 60). A cluster of eight genes, *esxA*, *esaA*, *essA*, *essB*, *esaB*, *essC*, *esaC*, and *esxB*, related to type VII secretion system [78] was present in the 15 *S. aureus *strains. Of the eight genes, *esxA*, *esaA*, *essA*, *essB*, *esaB*, and *essC *were present but *esaC *and *esxB *were absent in *S. aureus *MRSA252 and *S. lugdunensis *HKU09-01. *S. aureus *is known to carry a variety of mobile genetic elements such as prophages, plasmids, and transposons [2,72]. A total of 302, 166, and 27 protein families associated with phage, plasmid, and transposase were identified here. The numbers of protein families annotated as phage, plasmid, and transposase in *S. simiae *were 126, 75, and 13, whereas the numbers present in genomes of *S. aureus *ranged from 130-195, 84-124, and 11-20. This ranks *S. aureus *among the top of *Staphylococcus *genomes in terms of abundance of genes related to mobile genetic elements. Our results suggest that pathogenesis in the *S. aureus *group has developed by gene gain through horizontal transfer of mobile genetic elements, after divergence of *S. simiae *and *S. aureus *from their common ancestor.

## Authors' contributions

HS carried out the bioinformatics analyses, and wrote the manuscript. TL participated in the bioinformatics analyses. PPB performed the laboratory experiments. MJS conceived the study and helped write the manuscript. All authors read and approved the final manuscript.

## Supplementary Material

Additional file 1 **Supplementary Table S1**. Genomic information of the 28 *Staphylococcus *strains and *Macrococcus caseolyticus *JCSCS5402. **Supplementary Table S2**. Gene content table for the 28 *Staphylococcus *strains and *Macrococcus caseolyticus *JCSCS5402. The first 13 columns contain the protein family identification number, partial sequence (0, no; 1, one side; 2, both sides), amino acid length (Laa), locus_tag or protein_id (tag), functional annotations from different databases: COG, GenBank, JCVI, KEGG, VFDB, MvirDB, Pfam, and GO. The remaining columns show binary data (1 or 0) for presence or absence of each protein family for each of the 29 strains. **Supplementary Table S3**. Protein families present in *Staphylococcus aureus *and absent in other *Staphylococcus *species, and vice versa. The first 13 columns are explained in **Supplementary Table S2**. **Supplementary Table S4**. Protein families present in *Staphylococcus simiae *and absent in other *Staphylococcus *species, and vice versa. The first 13 columns are explained in **Supplementary Table S2**. **Supplementary Table S5**. Database categories that are over- or underrepresented in *Staphylococcus aureus *relative to *Staphylococcus simiae*. a = the number of the *S. aureus *strains' protein families in this category, b = the number of the *S. aureus *strains' protein families not in this category, c = the number of the *S. simiae *strain's protein families in this category, d = the number of the *S. simiae *strain's protein families not in this category, odds ratio = ad/bc, p-value obtained by Fisher's exact test, and q-value (false discovery rate adjusted p-value). Click here for file

Additional file 2 **Supplementary Figure S1**. A majority rule consensus of the maximum likelihood trees obtained from nucleotide sequences of the orthologous core genes for the 28 *Staphylococcus *strains and *Macrococcus caseolyticus *JCSCS5402 (outgroup). The percentages of genes that support the branches of the tree are indicated. Click here for file

Additional file 3 **Supplementary Figure S2**. A heatmap showing % identity values of TBLASTN (E-value cutoff of 1e-5) best hits in the 28 *Staphylococcus *strains and *Macrococcus caseolyticus *JCSCS5402, against *Staphylococcus *virulence genes deposited in Virulence Factors Database (VFDB). Click here for file
