# Supplementary figures and images for: Comparative genomic analysis of the genus Staphylococcus including Staphylococcus aureus and its newly described sister species Staphylococcus simiae
Source: BMC Genomics. 2012 Jan 24;13:38. doi: 10.1186/1471-2164-13-38 (PMC3317825; doi:10.1186/1471-2164-13-38)

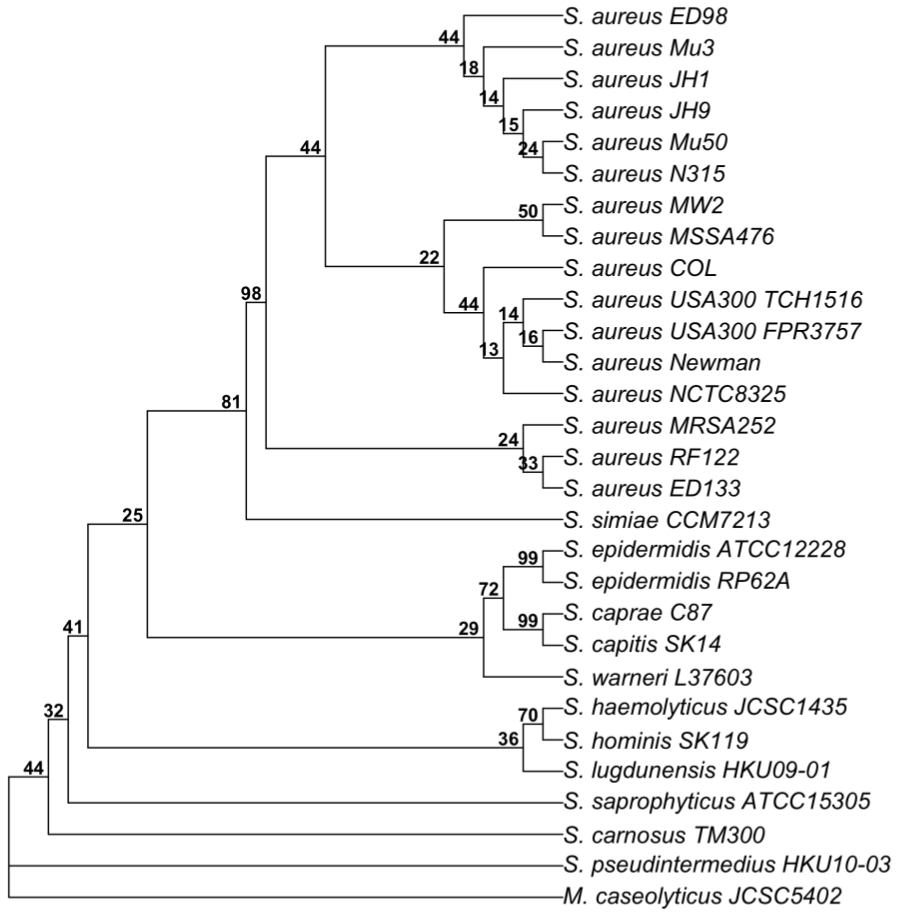

Supplement: Additional file 2 — Supplementary Figure S1. A majority rule consensus of the maximum likelihood trees obtained from nucleotide sequences of the orthologous core genes for the 28 Staphylococcus strains and Macrococcus caseolyticus JCSCS5402 (outgroup). The percentages of genes that support the branches of the tree are indicated. [file 1471-2164-13-38-S2.TIFF]

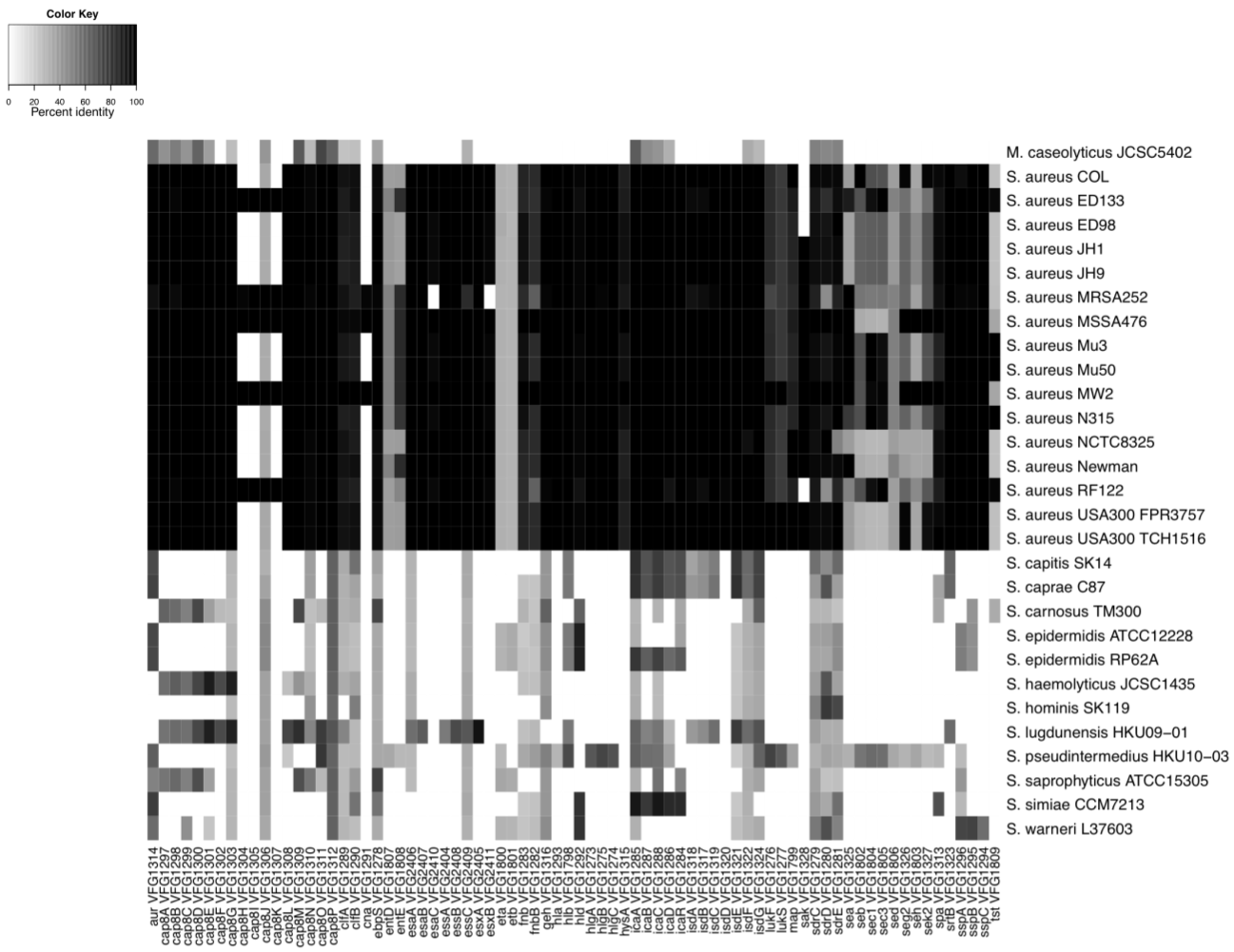

Supplement: Additional file 3 — Supplementary Figure S2. A heatmap showing % identity values of TBLASTN (E-value cutoff of 1e-5) best hits in the 28 Staphylococcus strains and Macrococcus caseolyticus JCSCS5402, against Staphylococcus virulence genes deposited in Virulence Factors Database (VFDB). [file 1471-2164-13-38-S3.TIFF]
